# Growth and Saxitoxin Production by *Cylindrospermopsis raciborskii* (Cyanobacteria) Correlate with Water Hardness

**DOI:** 10.3390/md11082949

**Published:** 2013-08-15

**Authors:** Ronaldo Leal Carneiro, Ana Beatriz Furlanetto Pacheco, Sandra Maria Feliciano de Oliveira e Azevedo

**Affiliations:** Institute of Biophysics Carlos Chagas Filho, Federal University of Rio de Janeiro, Rio de Janeiro 21949-902, RJ, Brazil; E-Mails: biafp@biof.ufrj.br (A.B.F.P.); sazevedo@biof.ufrj.br (S.M.F.O.A.)

**Keywords:** cyanobacteria, saxitoxins, paralytic shellfish poisoning, water hardness, ionic effect, growth

## Abstract

The cosmopolitan and increasing distribution of *Cylindrospermopsis raciborskii* can be attributed to its ecophysiological plasticity and tolerance to changing environmental factors in water bodies. In reservoirs in the semi-arid region of Brazil, the presence and common dominance of *C. raciborskii* have been described in waters that are considered hard. We investigated the response of a Brazilian *C. raciborskii* strain to water hardness by evaluating its growth and saxitoxin production. Based on environmental data, a concentration of 5 mM of different carbonate salts was tested. These conditions affected growth either positively (MgCO_3_) or negatively (CaCO_3_ and Na_2_CO_3_). As a control for the addition of cations, MgCl_2_, CaCl_2_ and NaCl were tested at 5 or 10 mM, and MgCl_2_ stimulated growth, NaCl slowed but sustained growth, and CaCl_2_ inhibited growth. Most of the tested treatments increased the saxitoxin (STX) cell quota after six days of exposure. After 12 days, STX production returned to concentrations similar to that of the control, indicating an adaptation to the altered water conditions. In the short term, cell exposure to most of the tested conditions favored STX production over neoSTX production. These results support the noted plasticity of *C. raciborskii* and highlight its potential to thrive in hard waters. Additionally, the observed relationship between saxitoxin production and water ion concentrations characteristic of the natural environments can be important for understanding toxin content variation in other harmful algae that produce STX.

## 1. Introduction

*Cylindrospermopsis raciborskii* is a filamentous diazotrophic cyanobacterium that is globally distributed in freshwater environments [1]. *C. raciborskii* blooms are increasing in occurrence and frequency, which is attributed to their invasive capacity [2,3,4,5]. This circumstance brings additional concern because some strains are toxic to water organisms and humans. Considering its worldwide occurrence, most characterized toxic strains produce cylindrospermopsin, a cytotoxic alkaloid [6]. However, toxic strains isolated from Brazilian water supplies produce neurotoxins associated with paralytic shellfish poisoning: saxitoxin (STX), neosaxitoxin (neoSTX), gonyautoxins (GTX1-4) and other STX analogues as well as other unidentified toxins [7,8,9]. These secondary metabolites are produced by freshwater prokaryotic cyanobacteria as well as by marine eukaryotic dinoflagellates. STX and its analogues (STXs) interfere with the function of voltage-gated ion channels, such as sodium, calcium and potassium channels, causing rapid neuromuscular paralysis [10,11].

The increasing and cosmopolitan distribution of *C. raciborskii* can be attributed to its ecophysiological plasticity, to the existence of diverse ecotypes with specific environmental adaptations and possibly to water temperature increases due to global climate change [2,12,13,14]. *C. raciborskii* thrives in tropical and temperate climates, illustrating its tolerance to changes in the water environment, such as the temperature, pH, light, conductivity, alkalinity and nutrient availability [2,3,15,16]. In general, the adaptive success of *C. raciborskii* is related to physiological traits, such as the ability to fix nitrogen, high affinity for ammonium and phosphorous, buoyancy control, and formation of akinetes [1,4,5]. 

*C. raciborskii* dominance is frequently observed in eutrophic and hypereutrophic reservoirs in the northeastern Brazilian semi-arid region, where high water conductivity values have been documented [17,18,19]. For example, *C. raciborskii* represented 12% of the phytoplankton biomass in reservoirs with a conductivity value of 19,630 μS [18] and dominated (>80%) the phytoplankton biomass in a reservoir with a conductivity value of 1000 μS [17]. The regional soils are rich in different carbonates (calcium, magnesium and sodium), which concentrate in waters due to run off and irrigation procedures [17]. In general, these reservoirs present conductivity values ranging from 300 to 3000 μS·cm^−1^ and alkalinity values from 300 to 5567 μEq·L^−1^ [18,19,20,21]. Alkalinity is routinely associated with water hardness and correlates with CaCO_3_ concentrations [22,23,24]. Waters with CaCO_3_ concentrations above 150 mg·L^−1^ or 1.49 mM are considered hard [24]. The carbonate concentrations in some reservoirs in Northeast Brazil can vary from 1.2 mM to 5 mM [25]. 

In field studies, physicochemical parameters, such as water salinity, conductivity and alkalinity, are typically assessed and their relationship with phytoplankton composition and dynamics presented [18,19,20]. However, such measurements are seldom accompanied by a description of the specific water composition that provides water hardness. Because *C. raciborskii* often dominates in hard waters in some Brazilian reservoirs, in this study, carbonate salts (CaCO_3_, MgCO_3_ or Na_2_CO_3_) were added to standard culture media to simulate increased water hardness. Growth and saxitoxin production of a *C. raciborskii* (T3) strain were evaluated. The corresponding chloride salts (CaCl_2_, MgCl_2_ or NaCl) were used as controls. The experimental results demonstrated that, after a shift to conditions simulating a hard water environment, the *C. raciborskii* (T3) strain responded sustaining growth as well as increasing STX cell quota. 

## 2. Results and Discussion

### 2.1. Growth in Different Conditions

To obtain a final concentration of 5 mM CO_3_^2−^, different carbonate salts, CaCO_3_, MgCO_3_ or Na_2_CO_3_, were added to ASM-1 medium. This CO_3_^2−^ concentration yielded different concentrations of the associated Ca^2+^, Mg^2+^, and Na^+^ cations in each case. Thus, to control for the presence of these cations, CaCl_2_, MgCl_2_ or NaCl was added to the ASM-1 media to obtain concentrations of 5 or 10 mM of each cation. 

Conductivity was measured in the media prior to cell inoculation, and values increased when the different salts were added. The addition of carbonate salts yielded values from 656 to 1401 μS, whereas the chloride salt values ranged from 808 to 2550 μS (Table 1). The lower conductivity increase observed for ASM-1 with CaCO_3_ in comparison to the control resulted from a partial precipitation of this salt.

**Table 1:** Growth medium and cell growth parameters of *C. raciborskii* (T3) cells cultivated in the presence of different salts.

Growth medium	Conductivity (μS)	pH ^a,b^	Exponential phase		Growth rate (μ) ^a^
			Duration	*R*^2^	
Control	568	7.43 ± 0.19	6	0.98	0.225 ± 0.03
CaCO_3_ 5 mM	656	7.98 ± 0.12 *	ND	0.92	0.100 ± 0.01 *
MgCO_3_ 5 mM	1196	8.66 ± 0.13 **	15	0.99	0.231 ± 0.02 **
Na_2_CO_3_ 5 mM	1401	8.76 ± 0.09 **	15	0.95	0.162 ± 0.01 **
CaCl_2_ 5 mM	1436	7.51 ± 0.24	ND	0.71	0.094 ± 0.02 *
MgCl_2_ 5 mM	1484	7.45 ± 0.35	ND	0.71	0.238 ± 0.02 *
NaCl 5 mM	808	7.55 ± 0.38	ND	0.75	0.197 ± 0.02
CaCl_2_ 10 mM	2550	7.55 ± 0.17	ND	0.34	0.029 ± 0.01 **
MgCl_2_ 10 mM	2475	7.45 ± 0.35	ND	0.86	0.235 ± 0.02 *
NaCl 10 mM	1350	7.53 ± 0.33	ND	0.89	0.104 ± 0.01 *

^a^ Defined as described in the experimental section; data are presented as the average ± standard deviation (*n* = 3); ^b^ pH values are the average of data from the 3rd to 15th day of sampling (*n* = 15); ND refers to exponential growth not detected by regression analysis (*R*^2^ < 0.95); in these cases, growth rate was calculated from day 0 to 15; Significant differences are reported in comparison to the control: * Dunn test, *p* < 0.05; ** Dunn test, *p* < 0.01.

Variations in pH were assessed during cellular growth and for each condition; the resulting values are presented as averages in Table 1. In media with added MgCO_3_ or Na_2_CO_3_, as expected, the pH values were higher than the control (Table 1), whereas no pH difference was observed in the other treatments compared to the control (Table 1). The salt was relatively insoluble in the CaCO_3_ treatment [24], and the small pH increase in comparison to other carbonate treatments was likely due to a partial precipitation of this salt during medium preparation. 

Considering the conductivity values and carbonate concentrations described for the reservoirs of the Brazilian semi-arid region where *C. raciborskii* is dominant, the set of modified media listed in Table 1 provided adequate salt concentrations and water hardness to study the response of the *C. raciborskii* strain to these conditions. 

**Figure 1:**
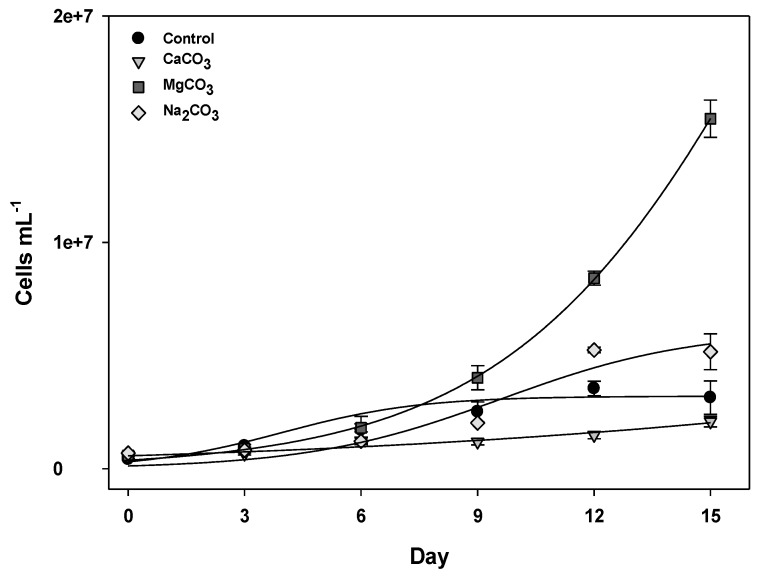
Growth curves of *C. raciborskii* (T3) cells exposed to calcium, magnesium or sodium carbonate salts (5 mM carbonate ion). The error bars represent the standard error (*n* = 3).

Only the control condition yielded observably different growth phases, where exponential growth occurred until the 6th day of culture (*R*^2^ = 0.98). Exponential growth phases were also determined for MgCO_3_ and Na_2_CO_3_ conditions (Table 1). In the other treatments, as an exponential growth phase was not apparent, the total growth rates were calculated considering differences in cell densities between the 15th day (*N*) and the inocula (*N*_0_) (Figure 1, Table 1).

Among the media supplemented with carbonate, MgCO_3_ was the most favorable condition for growth. In this case, cells grew exponentially until the 15th day of culture (Figure 1, Table 1). Na_2_CO_3_ addition slightly decreased the growth rate in comparison to the control, but the exponential growth phase still lasted for 15 days (as compared to 6 days in the control), thus providing a similar cell concentration after 15 days (Figure 1, Table 1). The opposite was observed for CaCO_3_, which continuously inhibited growth. Thus, in regard to the influence of water hardness on growth of *C. raciborskii* (T3), growth in hard water was not affected by the presence of CO_3_^2−^ but did change, either positively or negatively, depending on the combination of other cations. 

**Figure 2:**
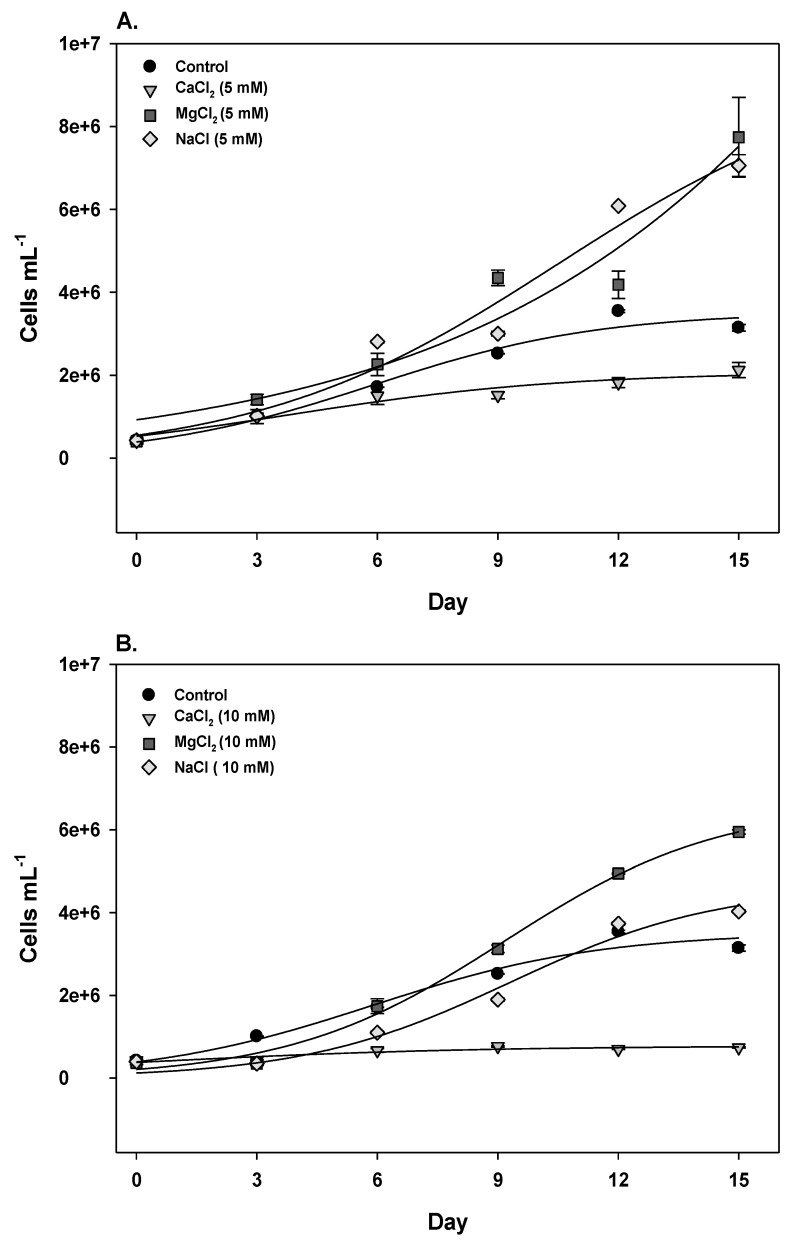
Growth curves of *C. raciborskii* (T3) cells exposed to calcium, magnesium or sodium chloride salts at 5 mM (**A**) or 10 mM (**B**). The error bars represent the standard error (*n* = 3).

When adding CO_3_^2−^, the respective cations were present at 5 mM (Ca^2+^, Mg^2+^) or 10 mM (Na^+^) in the control, and the same concentrations of these cations were tested as chloride salts. In these treatments, exponential growth was not observed (Table 1). MgCl_2_ stimulated the total growth rate for both tested concentrations, resulting in cell densities higher than the control after 15 days (Figure 2, Table 1). CaCl_2_ severely inhibited growth for both concentrations, which were the most unfavorable conditions tested (Figure 2, Table 1). The lower NaCl concentration (5 mM) did not affect the growth rate, whereas 10 mM NaCl lowered the growth rate compared to the control (Table 1). However, the cell density after 15 days exceeded that of the control (Figure 2), indicating a slow but sustained growth. Additionally, increasing the MgCl_2_ concentration from 5 to 10 mM negligibly affected growth, but a marked decrease was observed when NaCl (−47%) and CaCl_2_ (−69%) concentrations were increased.

A few studies have reported the influence of NaCl on *C. raciborskii* growth in culture conditions. Adding 10 mM NaCl to the *C. raciborskii* (T3) growth medium decreased growth (approximately 25%) after 6–7 days compared to the control [26]. Using another *C. raciborskii* strain isolated from a freshwater lake, tolerance to estuarine NaCl concentrations was tested [27]. The maximum NaCl concentration that supported growth was 69 mM, but optimum growth rates preserving CO_2_ fixation and nitrogenase activity were observed up to 34 mM. Based on environmental data, Calandrino & Pearl [28] subjected *C. raciborskii* to a high salinity condition (8.4 ppt) by adding NaCl and concluded that, although it was detrimental to growth and impaired biomass accumulation, the cells tolerated such a high salinity. Recently, with a *Raphidiopsis brokii* strain, a close phylogenetic relative of *C. raciborskii*, Soto-Liebe *et al*. demonstrated increased growth (measured by changes in Chl-a L^−1^ over 15 days) with 17 mM NaCl [29]. 

According to our observations, *C. raciborskii* (T3) growth was slow but not inhibited in the presence of sodium until 10 mM (Figures 1 and 2). As already suggested, this may have resulted from extra energy expenditure due to the activity of Na^+^/H^+^ antiporters to ensure appropriate intracellular concentrations of sodium [26].

The addition of calcium to the medium, either as CaCl_2_ or as CaCO_3_, severely inhibited *C. raciborskii* (T3) growth. This inhibition could have resulted from a direct influence of Ca^2+^ due to the charge to maintain Ca^2+^ homeostasis, which is similar to what has been suggested for Na^+^ stress [26]. The required activity of Ca^2+^ efflux mechanisms could explain the limited growth rate of cells maintained in high Ca^2+^ concentrations. Other factors may have contributed to growth inhibition, such as increased turbidity in the presence of CaCO_3_ due to Ca^2+^ precipitation, possibly together with another component, such as phosphate [30]. 

To our knowledge, the influence of magnesium on *C. raciborskii* growth has not previously been reported. The results in this study demonstrate a clear positive influence of Mg^2+^ on *C. raciborskii* growth. Mg^2+^ is a co-factor for many enzymes and a component of chlorophyll molecules. It mediates binding to phosphate compounds and carbon fixation [31,32], which explains its stimulatory influence on cellular growth.

In summary, our observations demonstrated that, following 15 days of exposure, magnesium promoted growth, sodium slowed but sustained growth, and calcium inhibited growth. Conductivity alone could not account for these observed differences, as exemplified by 10 mM MgCl_2_ and CaCl_2_, which conferred similar conductivity values but exerted opposite effects on growth (Table 1). This result indicates that altered growth profiles could be a specific response to some ions.

### 2.2. Saxitoxin Production

Saxitoxin (STX) and neosaxitoxin (neoSTX) production by *C. raciborskii* (T3) were quantified after six days of culture (exponential phase in control conditions) and after 12 days of culture (stationary phase in control conditions). As mentioned above, because the added salts differently affected *C. raciborskii* (T3) growth, cell samples at these different times cells were likely physiologically distinct. 

**Figure 3:**
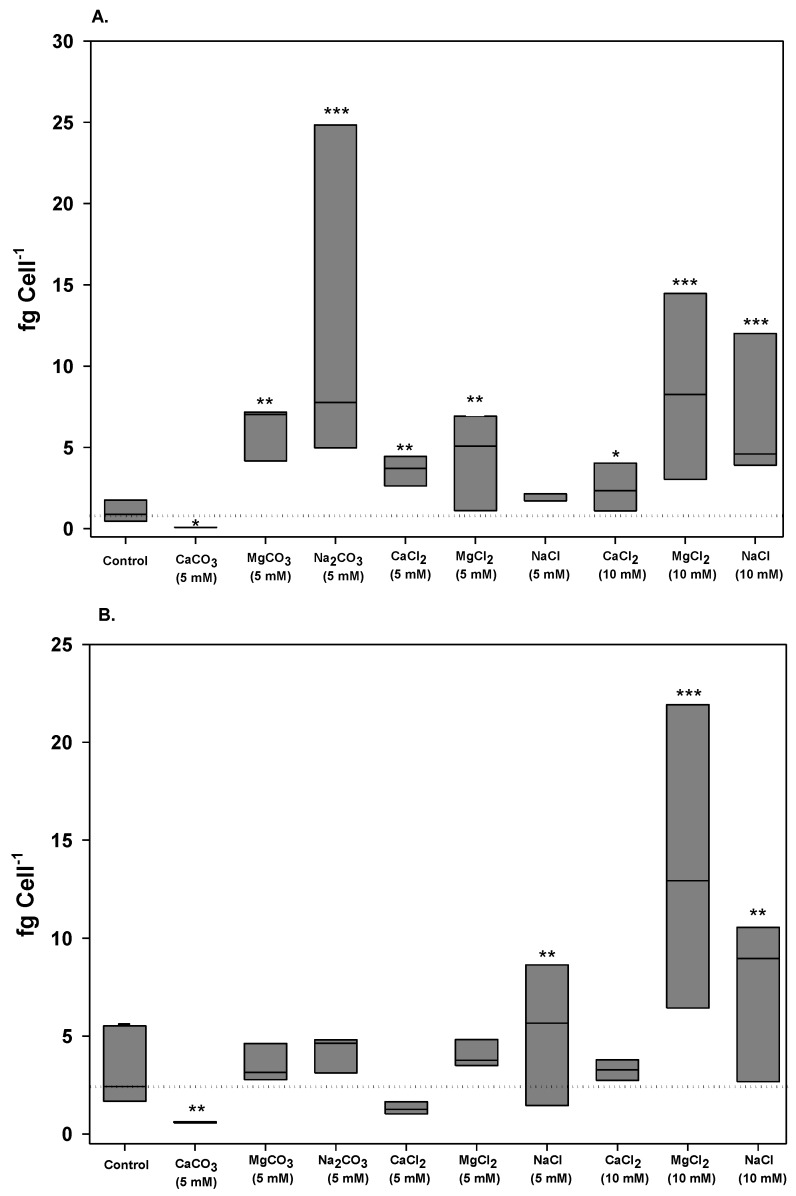
Box plot of variation of the total saxitoxin (STX + neoSTX) cell quota in *C. raciborskii* (T3) under different treatments. (**A**) Cell quota on the 6th day of culture; (**B**) Cell quota on the 12th day of culture. The dotted lines represent the median in the control condition. Significant differences are reported in comparison to the control: * Dunn test, *p* < 0.05; ** Dunn test, *p* < 0.01; *** Dunn test, *p* < 0.001.

**Figure 4:**
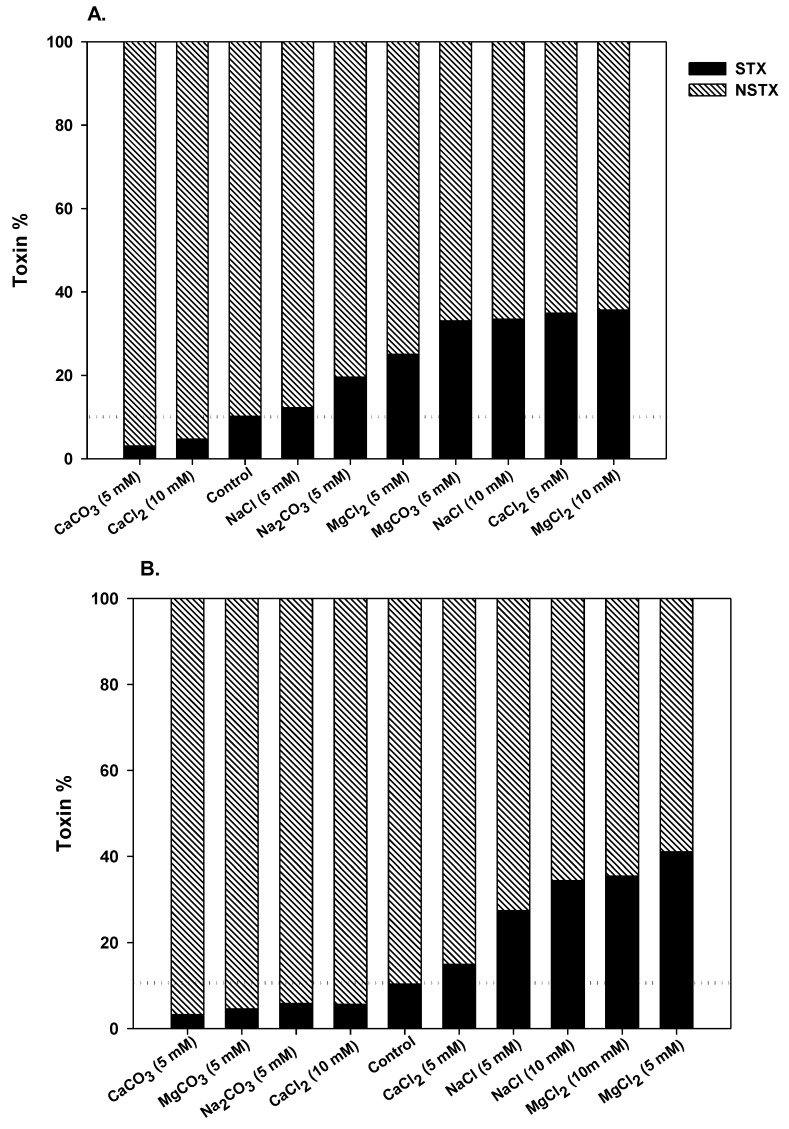
Contribution of saxitoxin and neosaxitoxin (%) to total saxitoxin production by *C. raciborskii* (T3) under different treatments. (**A**) 6th day of culture; (**B**) 12th day of culture. The dotted lines represent the saxitoxin percentage in the control condition.

The saxitoxin composition produced by the *C. raciborskii* (T3) strain has been described as neoSTX (87%), decarbamoyl-neoSTX (10%) and STX (3%) [33]. Thus, both STX and neoSTX represent 90% of the total toxin content in these cells. In this study, alterations in toxin profiles were analyzed in terms of total intracellular toxin content by adding the STX and neoSTX values and normalizing to the cell quota (fg cell^−1^) (Figure 3). We also assessed whether different treatments interfered with STX conversion by analyzing its variants (Figure 4 and Table 2). In all cases, extracellular STXs were not detected (values were below the detection limit of the method used: 15 ng·mL^−1^ for STX and 2 ng·mL^−1^ for neoSTX), which suggests that no measurable toxin was released at these sampling times. 

On the sixth day of culture, most tested treatments enhanced the total STX cell quotas compared to the control cultures. However, 5 mM NaCl yielded a STX cell quota similar to the control, and CaCO_3_ yielded the lowest STX cell quota (Figure 3). Na_2_CO_3_ and MgCl_2_ (10 mM) exerted the most positive influences on the total toxin cell quotas on the sixth day (Figure 3). 

After 12 days of culture, the general stimulatory influence of the treatments on toxin production was not maintained (Figure 3). Carbonate treatments did not stimulate toxin production in comparison to the control condition. Only NaCl and MgCl_2_ media exerted a positive influence. Increasing the concentrations of NaCl and MgCl_2_ to 10 mM also resulted in higher cellular toxin values. 

As we previously reported, the STX cell quota increased when *C. raciborskii* (T3) cells reach the stationary phase [34]. In this study, under control conditions, the total toxin content increased 3.1-fold from day 6 to day 12. The toxin content also varied during these times, some treatments increased and others decreased the total toxins cell quota on the 12th day in quite variable proportions (Table 2). These results indicate that the different treatments likely influence STX biosynthesis.

**Table 2:** Changes in saxitoxin content of *C. raciborskii* (T3) cells cultivated with salts at different sampling times.

Growth medium	Fold change from day 6 to 12 ^a^		
	Total	neoSTX	STX
Control	+3.1	+2.9	+5.6
CaCO_3_ 5 mM	+7.7	+7.7	+9.0
MgCO_3_ 5 mM	−1.7	−1.2	−13.0
Na_2_CO_3_ 5 mM	−3.0	−2.7	−6.8
CaCl_2_ 5 mM	−2.7	−2.1	−7.1
MgCl_2_ 5 mM	−1.1	−1.3	+1.44
NaCl 5 mM	+2.6	+2.1	+6.2
CaCl_2_ 10 mM	+1.3	+1.3	+1.6
MgCl_2_ 10 mM	+1.6	+1.6	+1.6
NaCl 10 mM	+1.1	+1.1	+1.1

^a^ Positive signal (+) refers to increases in the STX cell quota, and a negative signal (−) refers to decreases in the STX cell quota.

Separately, intracellular STX and neoSTX content of *C. raciborskii* (T3) also displayed significant differences between the different sampling times (6th day versus 12th day; Table 2). Despite the fact that CaCO_3_ greatly inhibited growth, the cells accumulated the most STX and neoSTX in comparison to other conditions after 12 days of culture, with a particularly high increase in STX content, whereas other carbonate treatments decreased the toxin content at this time (Table 2).

Evaluating total STXs as percentages, the neoSTX content was always higher than the STX content (Figure 4). However, the ratio of neoSTX to STX varied in different treatments (Figure 4, Table 2). In the control condition, STX comprised approximately 10% of the total STXs at both tested times. However, for most conditions, the STX content increased after 6 days of exposure, except for with CaCO_3_ and 10 mM CaCl_2_ (Figure 4). After 12 days, the STX content was higher than the control in the presence of chloride salts, but the opposite was observed for carbonate treatments. In some cases, the STX content increased to approximately 40% of the total saxitoxin content. Based on mouse bioassays, the relative STX toxicity can be equal to or higher than the neoSTX toxicity [35,36]. Thus, the observed shift in the toxin profile, favoring STX over neoSTX, in most tested conditions likely increased cell toxicity. 

To our knowledge, the influence of water hardness, represented by changes in conductivity or ionic composition, on *C. raciborskii* toxin cell quota in the natural environment has not been reported. In culture conditions, *C. raciborskii* (T3) growth in the presence of NaCl promoted the accumulation of STX coupled with an increase in cellular Na^+^ [26]. Concentrations of 5 or 10 mM NaCl resulted in a 24%–29% STX increase, but this influence was only tested for 2 h. These results were observed in media with a pH of 9.5, which requires Na^+^ transport for its maintenance. Therefore, the link between STX biosynthesis and cellular Na^+^ concentrations were suggested to be relevant to the maintenance of cellular homeostasis. Although, until now, this relationship remains hypothetical, our data agree with this hypothesis. Contrarily to the previous study [26], we evaluated the long term exposure to sodium in different salt forms. After 6 days, Na_2_CO_3_ resulted in the highest toxin content. The exposure of *C. raciborskii* to different sodium treatments would also demand an active ion transport to maintain cellular homeostasis, and STX synthesis could be part of this response. 

Recently, this idea was further extended in a study that examined the effect of NaCl (17 mM) on toxin production (gonyautoxins and STX) by *Raphidiopsis brookii* [29]. After 24 h of exposure, this condition initially decreased toxin synthesis, which was followed by a period of active release into the extracellular medium and then a decrease in both intra and extracellular levels. The authors suggested that toxin transport, through Na^+^/drug antiporters, can function as a protective mechanism against salt variation in the environment, adding to the ability of cells to adapt to a broad range of aquatic ecosystems. Because we analyzed STX variation after 6 or 12 days of culture in this study and did not detect STXs in the extracellular media, the possibility of a similar response by *C. raciborskii* T3 could not be examined.

Previously, an inhibitory effect of calcium chloride on *C. raciborskii* (T3) metabolism was reported [37]. Studying *C. raciborskii* saxitoxin biosynthesis *in vitro*, Kellman and Neilan [38] revealed that Ca^2+^ was inhibitory. Similarly, the present results demonstrated that calcium inhibited growth and yielded lower toxin contents than the other tested conditions. 

The addition of magnesium improved *C. raciborskii* (T3) growth and yielded toxin cell quotas that were higher or similar to the control (5 mM after 12 days of exposure). Thus, in contrast to calcium, the general stimulatory effect of magnesium on cellular metabolism was reflected in both enhanced growth and the toxin cell quota. 

In contrast to some studies, the aim of this work was to assess whether the presence of carbonates and the combined cations present in hard waters would affect *C. raciborskii* growth or saxitoxin production during long term exposure. To test this in culture, *C. raciborskii* (T3) cells, previously maintained in standard medium, were transferred to modified media without an acclimation period. After 6 days in most of the tested conditions, the total STX content was larger than in the control. However, this tendency did not persist later. The initial increase in saxitoxin production was likely a response to the environmental change, whereas the return to the original toxin content could represent an adaptation to long-term exposure. This was observed with MgCO_3_ and Na_2_CO_3_. 

In a semi-arid climate like Northeast Brazil, long-lasting drought periods typically cause intense evaporation leading to increased salt concentrations in the reservoirs. In addition, increased concentrations of carbonate salts as a result of run off or a rainy season can increase water hardness. Under such conditions, the risk for human exposure would be linked not only to the presence of toxic *C. raciborskii* cells but also to a cyanobacterial population with higher STX cellular quotas, especially where water is a crucial resource. Although monitoring the water quality to prevent the risk of human exposure to cyanotoxins is based on determining cellular density, water hardness is not included in the analysis even though it can affect toxin concentrations in periods concomitant with environmental change. The present results indicate that *C. raciborskii* can support varying water hardness, in accordance with its previously noted ecophysiological plasticity. Additionally, a new relationship between the production of saxitoxins and water with ions at concentrations characteristic of the natural environment was demonstrated, which can help understand the toxin variation in different cyanobacteria or other harmful algae that produce STX.

## 3. Experimental Section

### 3.1. Strain Maintenance and Growth Conditions

The *C. raciborskii* (T3) strain used in this study was isolated from the Billings reservoir in São Paulo State, Brazil [7]. This strain is maintained in the culture collection of the Laboratory of Cyanobacterial Ecophysiology and Toxicology (Federal University of Rio de Janeiro, Rio de Janeiro, Brazil). The T3 strain produces neoSTX, decarbamoyl-neoSTX and STX in decreasing order of relative abundance [33]. A non-axenic batch culture of this strain was maintained in ASM-1 medium [39] with aeration at 24 ± 2 °C with a 12 h light-dark cycle and a photon flux of 50 μmol photons m^−2^·s^−1^ (provided by common daylight fluorescent lamps). 

Cultivation in control conditions was performed in ASM-1 media, which contains 20 μM Ca^2+^, 40 μM Mg^2+^, 2.2 μM Na^+^ in different salts and no CO_3_^2−^ ion. We studied the influence of higher concentrations (5 mM) of carbonates salts on growth and toxin production of the *C. raciborskii* (T3) strain. Carbonate was added as CaCO_3_, MgCO_3_ or Na_2_CO_3_ to obtain a final concentration of 5 mM CO_3_^2−^. This CO_3_^2−^ concentration yielded different concentrations of the associated Ca^2+^, Mg^2+^, and Na^+^ cations in each case. Thus, the control for this cation concentration difference, ASM-1, was modified with CaCl_2_, MgCl_2_ or NaCl to obtain 5 mM or 10 mM concentrations of Ca^2+^, Mg^2+^ or Na^+^, respectively. Before inoculation, conductivity was measured using a conductivity meter. To obtain the inoculum, a T3 strain culture was maintained in standard conditions until stationary phase (approximately 12 days). Cultures with added salts were initiated with 5.0 × 10^5^ cells mL^−1^ and maintained in 3-liter glass flasks with 2 L of culture medium for 15 days; each condition was tested in triplicate. Aeration and light exposure were performed as described in the standard conditions. Sampling was performed every 3 days under aseptic conditions to assess the cellular concentrations, as described previously [34]. Growth rates were estimated according to Carneiro *et al*. [40]. Briefly, the exponential growth period was determined by exponential regressions, and the exponential growth phase was considered when the data adherence was higher than 95% (*R*^2^ ≥ 0.95). The growth rates were calculated using the exponential regression formula 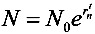 where *N* is the number of cells at time *t*, *N*_0_ is the initial number of cells and *r_n_* is the growth rate [30]. The growth curves were fitted for cell number data to non-linear functions using the logistic curve model as described by Soares *et al*. [41], where the biovolume was replaced by cells mL^−1^. 

### 3.2. STX and neoSTX Analysis

To determine STX and neoSTX concentrations, 500 mL samples were harvested on the 6th day (exponential phase in the control) and 12th day (stationary phase in the control), filtered on borosilicate filters (45 mm diameter) (Millipore, Billerica, MA, USA), and the filters and filtrated material were stored at −20 °C. HPLC analysis was performed within 24 h of the extraction procedure. The toxins were extracted as previously described [34] and were analyzed in both intracellular and extracellular fractions. Decarbamoyl-neoSTX was not quantified due to a lack of an appropriate standard. 

STX and neoSTX were analyzed according to Oshima [42] using a Shimadzu HPLC system with a silica-base reversed phase column (125 mm × 4.0 mm, 5 μm; Lichrospher 100 Reversed Phase 18). The chromatographic condition consisted of a mobile phase of 2 mM heptanesulfonate in 30 mM ammonium phosphate and 6% acetonitrile, pH 7.1. The toxins were detected using a fluorometric detector with an excitation at 330 nm and an emission at 390 nm. The toxins were identified and quantified by comparison with known retention times and integrated areas of STX and neoSTX standards purchased from the Institute of Marine Bioscience, National Research Council of Canada (Halifax, NS, Canada).

### 3.3. Statistical Analysis

The data were expressed as the mean values ± standard error (SE). The data for each experimental variable were tested for normality. Differences among the standard deviations were determined using Barlett’s test. Normality was tested using Kolmogorov and Smirnov’s test. If the data were classified as nonparametric, a one-way analysis of variance (ANOVA) was performed using the Kruskal-Wallis (KW) test for multiple comparisons. The post-hoc Dunn’s test was used to assess the specific differences between two treatments. All tests were performed with a significance of 95% (*p* < 0.05) using the GraphPad Instat 3.0 software.

## 4. Conclusions

This study tested the ability of a *C. raciborskii* saxitoxin producer strain to thrive in increasingly hard water. The results demonstrated that this strain tolerates this condition, which is consistent with the current idea that *C. raciborskii* exhibits a significant physiological plasticity. In the short term, cell exposure to most of the tested conditions increased saxitoxin production and favored STX production over neoSTX production. Finally, we suggest that water hardness should be considered in combination with cell density as a potential factor that could increase the risk of human exposure to STXs. 
